# Immune Profiling Identifies High-Risk Neutrophil-Rich Subtype in Checkpoint Inhibitor Nephritis

**DOI:** 10.1016/j.ekir.2025.103766

**Published:** 2026-01-06

**Authors:** Idris Boudhabhay, Helene Lazareth, Julie Oniszczuk, Mathilde Burki, Olivier Aubert, Alaeddine Redissi, Hamza Sakhi, Elsa Ferriere, Elizabeth Fabre, Francesca Lucibello, Nicolas Girard, Anne Grunenwald, Houcine Hamidi, Emma Fleury, Marie Agnes Dragon-Durey, Florence Runyo, Camille Cohen, Stephanie Chhun, Nicolas Veyrenche, Anne Jamet, Jacques Fourgeaud, Béatrice Regnault, Philippe Pérot, Marie-Sophie Meuleman, Celine Mayinga, Alexandre Karras, Jean-Paul Duong Van Huyen, Marion Rabant, Lubka T. Roumenina, Pierre Isnard

**Affiliations:** 1 Centre de Recherche des Cordeliers, Institut National de la Santé et de la Recherche Médicale, Sorbonne Université, Université de Paris Cité, Team Inflammation, Complement and Cancer, Paris, France; 2 Department of Nephrology and Kidney Transplantation, Necker Hospital, Assistance publique - Hôpitaux de Paris (AP-HP), Paris, France; 3 Paris Cité University, Paris, France; 4 Groupe de Recherche Interdisciplinaire Francophone en Onco-Néphrologie, Paris, France; 5 University Hospital Federation COMET, Paris, France; 6 Department of Nephrology, European Hospital Georges Pompidou, AP-HP, Paris, France; 7 Department of Nephrology, Foch Hospital, Suresnes, Paris, France; 8 Department of Nephrology and Kidney Transplantation, Felix Guyon Hospital, Saint-Denis, La Réunion, France; 9 Department of Thoracic Oncology, European Hospital Georges Pompidou, AP-HP, Paris, France; 10 Institut Curie, Institut du Thorax, Paris, France; 11 Department of Nephrology and Hemodialysis, CHI de Poissy-St Germain en Laye, Poissy, France; 12 Department of Nephrology and Hemodialysis, Hôpital Bichat - Claude Bernard, AP-HP, Université Paris Cité, Paris, France; 13 Laboratory of Immunology, Hôpital Necker-Enfants Malades, AP-HP, Centre-Université de Paris, Paris, France; 14 Department of Clinical Microbiology, Necker-Enfants Malades Hospital, AP-HP Centre Université de Paris Cité, Paris, France; 15 Pathogen Discovery Laboratory, Institut Pasteur, Université Paris Cité, Paris, France; 16 Department of Pathology, Necker Hospital, AP-HP, Paris, France; 17 INSERM U1151, CNRS UMR8253, Institut Necker Enfants Malades, Paris, France

**Keywords:** acute interstitial nephritis, checkpoint inhibitors, complement, immune phenotyping, immunotherapy, neutrophil

## Abstract

**Introduction:**

Immune checkpoint inhibitor (ICI)-induced acute interstitial nephritis (AIN) (ICI-AIN) is the leading cause of ICI-associated acute kidney injury (AKI). ICI-AIN is characterized by mononuclear immune infiltrates, although the mechanisms behind their toxicity remain unclear. We aimed to characterize these infiltrates in kidney biopsies and assess their correlation with clinical outcomes and therapeutic responses.

**Methods:**

We retrospectively analyzed 49 biopsy-proven ICI-AIN cases using multiplex immunofluorescence to quantify immune cells (macrophages, neutrophils, B cells, T cells, and plasmocytes). Unsupervised clustering was used to identify patient groups, which we then correlated with clinical presentation and outcomes. Finally, we explored the role of C5a/C5aR1 in neutrophil recruitment.

**Results:**

Unsupervised clustering revealed 3 immune phenotypes as follows: (i) low mononuclear (cluster 1), (ii) high mononuclear (cluster 2), and (iii) neutrophil-rich (cluster 3). Cluster 3 was associated with higher systemic inflammation (C-reactive protein: 84 vs. 15–24 mg/l, *P* = 0.0002; neutrophil-to-lymphocyte ratio (NLR): 7 vs. 3.2–2.3, *P* < 0.0001) and more severe initial AKI (peak creatinine: 360 vs. 215–208 μmol/l, *P* = 0.0001). Histologically, it was marked by granular casts and neutrophilic tubulitis (*P* < 0.0001). Despite the pyelonephritis-like appearance, urine cultures and metatranscriptomic analysis both ruled out infection. At 12 months, renal response rates to steroids were 93% (cluster 2), 67% (cluster 1), and 38% (cluster 3) (*P* = 0.004). Relapses occurred more frequently in cluster 3 (38% vs. 11% in cluster 1, 0% in cluster 2, *P* = 0.01). Urine C5a correlated with C5aR1+ neutrophil infiltration (rho = 0.78).

**Conclusion:**

Our findings identify distinct ICI-AIN subtypes, with a neutrophil-rich cluster linked to complement activation and poor prognosis, offering insights into refining diagnosis and treatment strategies.

## Graphical abstract

**Figure:**
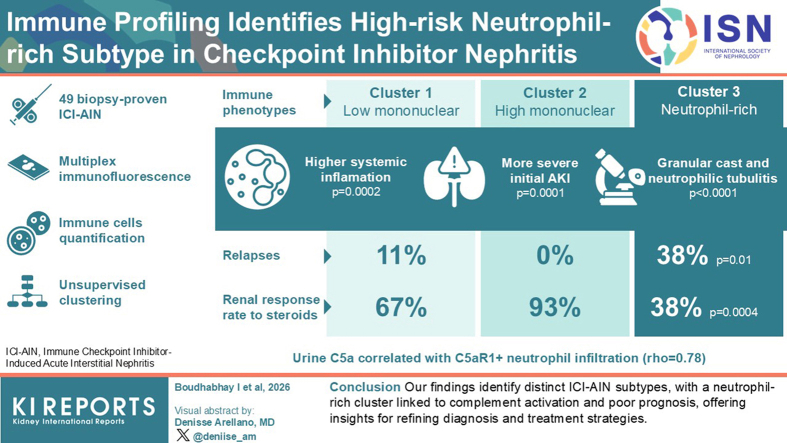

ICIs have revolutionized cancer treatment by enhancing antitumor immunity through the blockade of PD-1, PD-L1, and CTLA-4.^1^^,^^2^ However, this potent immune activation comes at the cost of immune-related adverse events (irAEs), including renal complications that affect approximately 2% to 5% of treated patients.^3^, ^4^, ^5^, ^6^ Among these, AIN is the most common, often causing severe AKI and reduced survival, especially in patients with incomplete renal recovery.^7^, ^8^, ^9^, ^10^, ^11^, ^12^ Despite its clinical importance, the underlying immune mechanisms driving ICI-AIN remain poorly characterized.^13^, ^14^, ^15^ More importantly, these severe complications may lead to discontinuation or modification of anticancer treatment strategies, that may affect patient outcome.^6^ Thus, gaining insight into the pathophysiology of ICI-AIN may improve diagnosis, treatment, and cancer management while minimizing serious side effects.

Histological studies of ICI-AIN reveal T-cell–, B-cell–, and macrophage-dominated interstitial infiltrates, primarily focusing on biomarker identification rather than distinguishing patient subgroups by immune cell composition.^14^, ^15^, ^16^ We hypothesized that immune infiltrate composition in kidney biopsies could shape the clinico-biological presentation, treatment response, and renal outcome in patients with ICI-AIN.

By using multiplex immunofluorescence and hierarchical clustering, we revealed distinct patient groups with the following: (i) divergent clinico-biological features at ICI-AIN diagnosis and (ii) varying treatment responses, highlighting immune mechanism heterogeneity. Notably, we identified a neutrophil-enriched subgroup resembling acute pyelonephritis, linked to C5a/C5aR1 axis of complement activation. Overall, our results provide novel insights into the immune landscape of ICI-AIN and unveil previously unrecognized pathways that could inform new approaches for diagnosis and treatment.

## Methods

### Study Design

Kidney biopsies referred for first-line histopathological diagnosis to the Paris-Cité Nephropathology department (Necker Hospital) between January 1, 2017, and July 31, 2024, were screened for ICI-AIN cases. Referrals came from Necker and Pompidou (Paris), Foch (Suresnes), Poissy (Poissy), and Felix Guyon (Saint-Denis, La Réunion) hospitals.

### Study Approval

This study complies with the 2000 Declaration of Helsinki. Written informed consent was obtained from patients, and the protocol was approved by the local ethics committee.

### Patients

Patients aged ≥ 18 years with biopsy-proven ICI-AIN were included. Exclusion criteria were kidney transplant, inadequate biopsy (< 8 glomeruli), incomplete medical records at biopsy, or < 90 days of follow-up. Patients were followed-up with from the biopsy date (index date) until dialysis initiation, death, or data cutoff (before July 31, 2025). Clinical and biological data were retrospectively extracted from medical records. Clinical variables included sex (collected as a biological variable, male/female, from medical records), age, history of hypertension, diabetes, chronic kidney disease, cancer type, and concurrent use of AIN-inducing drugs (proton pump inhibitors, nonsteroidal antiinflammatory drugs, and/or antibiotics). In addition, nephrotoxic chemotherapy use, ICI type, and presence of other irAEs were recorded. Baseline serum creatinine (SCr) was the value closest to ICI initiation (up to 1 year prior). Baseline NLR was calculated before ICI initiation. At biopsy, peak SCr (within 2 weeks), urinary protein-to-creatinine ratio, urinary sediment, antinuclear antibodies, C-reactive protein, eosinophil, lymphocyte, and neutrophil counts were recorded. NLR and urinary CXCL9 were calculated.^17^, ^18^, ^19^ Follow-up laboratory results included SCr and estimated glomerular filtration rate, using the Chronic Kidney Disease–Epidemiology Collaboration without race correction and NLR.^20^

### Inclusion Criteria

Patients were included if they met all 3 criteria as follows: (i) AKI attributed to ICI by the treating physician, (ii) ≥ 50% increase in SCr from baseline, and (iii) biopsy-confirmed AIN as per previous studies.^10^^,^^11^ Patients who had received corticosteroids prior to kidney biopsy were not included.

### Renal Outcomes

Complete recovery was defined as SCr returning to < 31 μmol/l (0.35 mg/dl) above baseline. Partial recovery was SCr > 31 μmol/l but < 2× baseline, or renal replacement therapy discontinuation regardless of SCr, as previously described.^10^^,^^21^ Recurrent ICI-AIN was defined as a ≥50% SCr increase from the poststeroid baseline, attributed to ICI by the treating physician.^10^^,^^11^

### Kidney Biopsies

Kidney biopsies routinely included 1 fragment fixed in formalin-alcohol-acetic acid and paraffin-embedded, and 1 frozen fragment. The fixed tissue was sectioned at 4 μm for hematoxylin and eosin, periodic acid–Schiff, Masson’s trichrome, and methenamine silver staining. Two kidney pathologists (PI and MR) blinded to clinical data, evaluated lesions. The frozen fragment, routinely obtained for immunofluorescence, was used for eosinophil quantification after hematoxylin and eosin staining and, when available, for metagenomic analyses. Lesions were graded per the Banff Classification, including interstitial inflammation, tubulitis, peritubular capillaritis, interstitial fibrosis, tubular atrophy, fibrous intimal thickening, and arteriolar hyalinosis.^22^ Acute tubular injury was scored semiquantitatively based on the proportion of affected tubules or interstitial area: absence (0%–10%, 0), mild (10%–25%, 1), moderate (25%–50%, 2), and severe (> 50%, 3). Histologic characteristics used to identify acute tubular injury were epithelial cell flattening or simplification, necrotic or apoptotic cells within the tubular section or lumen, detached epithelial cells forming casts, and interstitial edema. Granular casts were defined as neutrophil-containing casts in tubular lumens, quantified as the highest count per 20× field (approximately 1 mm^2^). Eosinophils were averaged per 20× field across the entire frozen section. Immunofluorescence was performed on frozen kidney biopsies using antibodies against IgA, IgG, IgM, kappa, lambda, C3, C1q, and fibrinogen with the BOND-III automated stainer (Leica Biosystems). For immunohistochemistry, 4 μm paraffin sections underwent antigen retrieval and were stained on the BOND-III using rabbit polyclonal anti-CD3 (Dako, 1:200), mouse monoclonal anti-CD20 (Dako, 1:400), anti-CD68 KP1 (Dako, 1:3000), and anti-CD15 (Leica Biosystems).

### Multiplexed Immunofluorescence

Multiplex immunofluorescence was performed on 4 μm formalin-fixed paraffin-embedded sections using the BOND Rx stainer (Leica). Antibodies targeted CD68 (macrophages), CD66b (neutrophils), CD3 (T cells), CD20 (B cells), MUM1 (plasmocytes), CD34 (endothelial cells), and C5aR1. Antirabbit/mouse/rat HRP polymers and TSA Opal fluorophores were used for detection (Supplementary Table S1). Slides were counterstained with DAPI (Akoya Biosciences) and cover slipped. A spectral library was created using single-antibody stains and autofluorescence controls. Slides were scanned on the Vectra Polaris System (Akoya) and unmixed using InForm Tissue Studio v3.0. Quantification was performed with Halo software (Indica Labs); cells were segmented via DAPI, and glomeruli were automatically excluded from the analysis using CD34 staining to delineate glomerular capillary loops. Immune cell densities (cells/mm^2^) were calculated from cell counts and area analyzed. Unsupervised hierarchical clustering and heatmaps were generated using the R package (R studio software) *pheatmap*, with Euclidean distance for patients (rows), correlation distance for cell types (columns), and Ward.D linkage to minimize intra-cluster variance.

### Complement Activation Fragments in Urine

We applied our recently developed “humoral Complementomics” approach^23^^,^^24^ adapted for urine, on 19 cohort samples from biocollection DC-2009-955 (IRB 2011-100073_38) in comparison with 17 healthy donors (9 males and 8 females, aged 25–60 years, with no known history of renal disease and not taking any regular medication) and 10 patients treated with ICI without AKI (ICI-noAKI, clinical data in Supplementary Table S2). C4d and C3d were measured using enzyme-linked immunosorbent assay (Svar Life Science; Hycult Biotech), and a MicroVue Complement Multiplex panel (QuidelOrtho) was used to detect Ba, Bb, C3a, C5a, sC5b-9, C4a, and Factor H (7-plex HQ1M221128 19723 001). Samples were diluted 1:100, and all enzyme-linked immunosorbent assays were run simultaneously to avoid freeze–thaw cycles. Chemiluminescence signals were captured using the Q-View Imager LS and analyzed with Q-View Software. Results were normalized to urine creatinine.

### Metatranscriptomics

Nucleic acids were extracted from nitrogen-frozen and OCT-embedded kidney biopsies using the MagNA Pure Compact RNA Isolation Kit (Roche) per manufacturer’s protocol. Metatranscriptomic analyses were performed as previously described.^25^ A detailed description of the methods is provided in the Supplementary Methods.

### Statistical Analyses

Qualitative variables were expressed as frequencies and compared using Fisher exact test. Quantitative variables were reported as medians with interquartile ranges and compared using the Mann–Whitney U test (2 groups) or Kruskal–Wallis test (3 groups). Dunn’s test with Bonferroni correction was applied for *post hoc* comparisons. Spearman’s rank correlation was used to assess associations. For time-to-event analyses, we used Kaplan–Meier curves and log-rank tests, specifically for AKI onset post-ICI, steroid response, and ICI-AIN relapses. Analyses were performed with R (v4.3.2) and GraphPad Prism (v10).

## Results

### Study Population and Baseline Characteristics

Between January 1, 2017, and July 31, 2024, 61 kidney biopsies were initially referenced as ICI-AIN at the Necker Pathology Department. Iterative biopsies from the same patients (*n* = 3), alternative diagnoses (*n* = 2), and patients without follow-up data (*n* = 2) were excluded, as well as 5 cases with inadequate biopsy material. Ultimately, 49 index cases with a biopsy-proven diagnosis of ICI-AIN were included in the study (Supplementary Figure S1).

Baseline clinical and biological characteristics are listed in Table 1. The median age was 65 (57–76) years, with 22 patients (45%) being female. Baseline estimated glomerular filtration rate at ICI initiation was 86 (67–99) ml/min per 1.73 m^2^, and 9 patients (18%) had preexisting chronic kidney disease of at least stage 3. ICIs were primarily used to treat nonsmall cell lung carcinoma in 25 patients (51%). Concomitant nephrotoxic chemotherapy was used in 28 (57%) of the patients and proton pump inhibitor in 43% of the cases. Thirty-seven patients (76%) experienced at least a partial oncologic response under ICI therapy.

**Table 1:** Baseline characteristics and immune checkpoint inhibitor–induced acute interstitial nephritis events

	Whole cohort *N* = 49	Cluster 1 *n* = 18	Cluster 2 *n* = 15	Cluster 3 *n* = 16	*P*-value^a^
Female, *n* (%)	22 (45)	8 (44)	7 (47)	7 (44)	0.9
Age,^b^ yrs, median (IQR)	65 (57–76)	65 (58–73)	68 (62–80.0)	63 (53–77)	0.5
Comorbidities, *n* (%)					
Diabetes	11 (22)	4 (22)	4 (33)	3 (19)	0.6
AHT	15 (31)	5 (36)	6 (50)	4 (25)	0.4
CKD ≥ 3	9 (18)	3 (18)	2 (13)	4 (25)	0.3
Baseline SCr, μmol/l, median (IQR)	77 (65–92)	77 (65–92)	84 (65–94)	70 (59–97)	0.9
Baseline eGFR,^c^ (ml/min per 1.73 m^2^), median (IQR)	86 (67–99)	84 (68–99)	84 (68–93)	88 (57–99)	0.7
Baseline neutrophil-to-lymphocyte ratio	3.9 (2.8–5.6)	3.4 (2.7–4.8)	2.9 (2.1–4.2)	5.7 (3.4–8.5)	0.005
Malignancy, *n* (%)					0.4
NSCLC	25 (51)	9 (50)	5 (33)	11 (69)	
Genitourinary	10 (20)	4 (22)	3 (20)	3 (19)	
Melanoma	3 (6)	1 (6)	2 (13)	0 (0)	
Digestive	3 (6)	2 (11)	1 (7)	0 (0)	
HNSCC	3 (6)	0 (0)	2 (13)	1 (6)	
Breast	4 (8)	2 (11)	1 (7)	1 (6)	
Mesothelioma	1 (2)	0 (0)	1 (7)	0 (0)	
PPI,^d^*n* (%)	21 (43)	7 (39)	7 (47)	7 (44)	0.6
Concomitant nephrotoxic chemotherapy,^e^*n* (%)	28 (57)	12 (67)	7 (47)	9 (56)	0.5
Response to ICI therapy, *n* (%)					0.9
CR/PR	37 (76)	13 (72)	11 (73)	13 (81)	
Stable disease	11 (22)	4 (22)	4 (27)	3 (19)	
Progression	1 (2)	1 (6)	0 (0)	0 (0)	
ICI class, *n* (%)					> 0.9
Anti-PD1	47 (96)	17 (94)	15 (100)	15 (94)	
Anti-PD-L1	2 (4)	1 (6)	0 (0)	1 (6)	
Combo anti-CTLA4 + anti-PD1/PD-L1	3 (6)	2 (11)	1 (7)	0 (0)	
ICI-AIN events^f^					
Delay between ICI initiation & AKI,^g^ wks, median (IQR)	14 (9–24)	17 (12–39)	15 (9–23)	11 (6–21)	0.08
Extrarenal irAEs, *n* (%)	12 (24)	5 (28)	3 (20)	4 (25)	0.9
Peak SCr, μmol/l, median (IQR)	256 (191–358)	215 (187–263)	208 (165–258)	360 (271–557)	0.0001
UPCR, g/g	0.4 (0.3–0.9)	0.35 (0.2–0.6)	0.3 (0.3–0.5)	1 (0.4–1.6)	0.01
Leukocyturia, WBC/μl, median (IQR)	17 (1–65)	6 (0–50)	18 (1–65)	17 (1–65)	0.6
Neutrophil-to-lymphocyte ratio, median (IQR)	3.8 (2.3–6.5)	3.2 (1.9–4.6)	2.3 (2.1–4.5)	7.0 (5.3–8.3)	< 0.0001
Peak CRP (mg/l), median (IQR)	30 (15–77)	15 (3–48)	24 (16–70)	84 (39–131)	0.0002
Blood eosinophil count,^b^ G/l, median (IQR)	0.1 (0.02–0.20)	0.03 (0.01–0.2)	0.04 (0.01–0.2)	0.1 (0.02–0.2)	0.4
Positive ANA, *n* (%)	17 (35)	6 (33)	6 (40)	5 (131)	0.8

AHT, arterial hypertension; AKI, acute kidney injury; ANA, antinuclear antibodies; CKD, chronic kidney disease; CR, complete response; CRP, C-reactive protein; CTLA-4, cytotoxic T lymphocyte-associated antigen 4; eGFR, estimated glomerular filtration rate; HNSCC, head and neck squamous cell carcinoma; ICI, immune checkpoint inhibitor; ICI-AIN, ICI-induced acute interstitial nephritis; IQR, interquartile range; irAEs, immune-related adverse events; NSCLC, nonsmall cell lung cancer; PD-1, programmed cell death 1; PD-L1, programmed death-ligand 1; PPI, proton pump inhibitor; PR, partial response; SCr, serum creatinine; UPCR, urine-protein-to-creatinine ratio; WBC, white blood cells.

Data are shown as median (IQR) or *n* (%).

All data are complete.

a The *P*-value corresponds to the comparison between patients in the 3 clusters (not the entire cohort), using the Kruskal-Wallis test for quantitative variables and Fisher exact test for qualitative variables.

b At the time of kidney biopsy.

c Baseline eGFR calculated based on CKD-Epidemiology Collaboration equation without race correction.^17^

d PPIs were assessed in the 30 days preceding ICI-AIN.

e Concomitant chemotherapies were assessed in the 30 days preceding ICI-AKI.

f At the time of kidney biopsy (+/- 1 wk).

g The initiation of AKI was defined as the first date when the creatinine value exceeded the baseline by ≥50%.

### Patients Clustering by Immune Cell Infiltration

**Figure 1:**
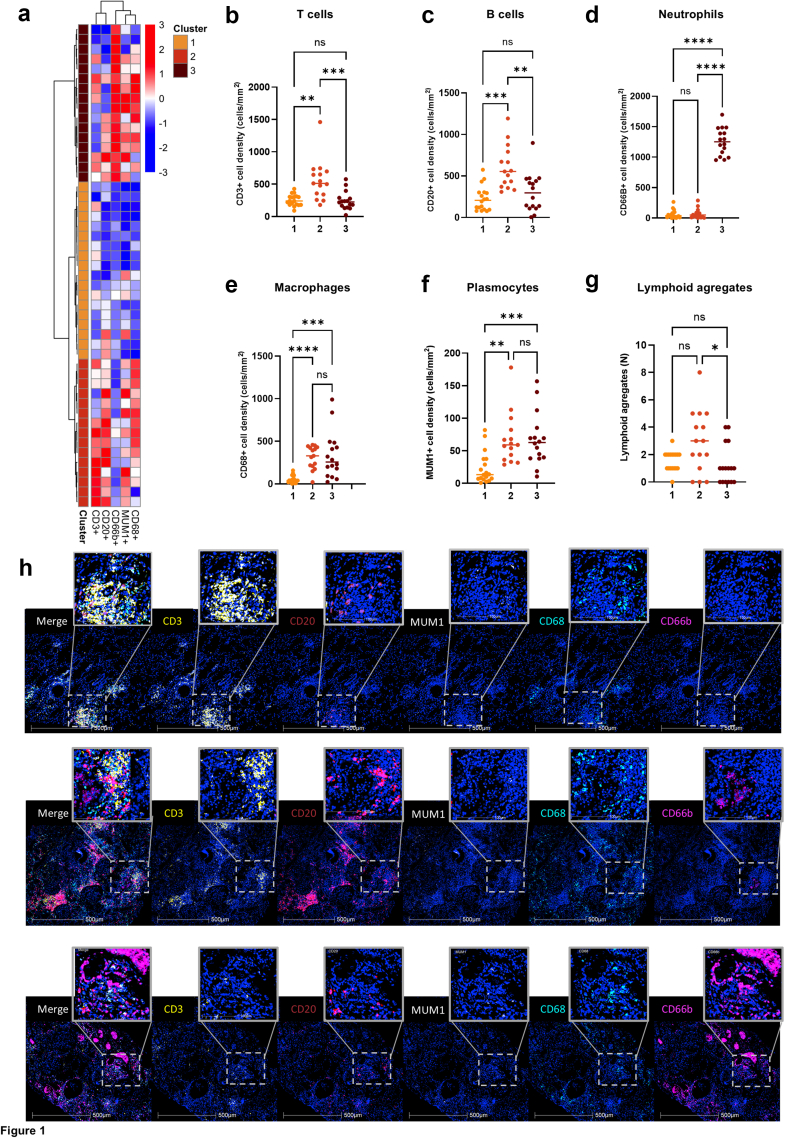
Immune phenotyping of patients with immune checkpoint inhibitor–induced acute interstitial nephritis identifies 3 distinct clusters. (a) Heatmap with row-level Z-score normalization depicting immune cell densities across samples. Unsupervised hierarchical clustering (Ward’s method) identified 3 distinct immune profiles (dendrogram, left). Comparison of cell densities across the 3 clusters: (b) T cells (CD3+), (c) B cells (CD20+), (d) neutrophils (CD66b+), (e) macrophages (CD68+), (f) plasmocytes (MUM1+), and (g) the relative number of lymphoid aggregates per biopsy. (h) Representative images of different immune cell types in the 3 clusters. Color code: T cells (white), B cells (red), plasmocytes (yellow), macrophages (cyan), and neutrophils (magenta). ∗*P* ≤ 0.05; ∗∗*P* < 0.01; ∗∗∗*P* < 0.001; ∗∗∗∗*P <* 0.0001.

To precisely characterize the immune infiltrate in patients with biopsy-proven ICI-AIN, we performed multiplex immunofluorescence using the following markers: CD68 (macrophages), CD66b (neutrophils), CD3 (T lymphocytes), CD20 (B lymphocytes), and MUM1 (plasma cells). Overall, the interstitial immune infiltrate consisted mainly of T and B cells, along with macrophages whereas neutrophils and plasma cells were less frequent (Supplementary Table S3). Interestingly, lymphoid aggregates, potentially indicative of tertiary lymphoid structures, were frequently observed, present in 39 (80%) of the analyzed kidney biopsies, with a median number of 2 (1–3) lymphoid aggregates per kidney biopsy, as previously described^13^ (Supplementary Table S3 and Supplementary Figure S2). To identify patient groups with similar immune infiltrate patterns, we performed unsupervised hierarchical clustering using the Ward method to analyze immune cell populations. We identified the following 3 distinct clusters of patients characterized by different immune cell densities (Figure 1a): (i) cluster 1, defined as low mononuclear cell infiltrate was characterized by a significantly reduced number of macrophages (*P* < 0.0001 and *P* = 0.0001 compared with clusters 2 and 3, respectively) and plasma cells (*P* = 0.002 and *P* = 0.0007 compared with clusters 2 and 3, respectively) as compared to the other 2 clusters and by less lymphocytes (B and T cells) as compared with cluster 2 (*P* = 0.0001 and 0.003, respectively); (ii) cluster 2, defined as high mononuclear cell infiltrate was characterized by significantly more T cells than the other 2 clusters (*P* = 0.003 and 0.0005 compared with cluster 1 and 3, respectively) as well as more B cells (*P* = 0.0001), macrophages (*P* < 0.0001) and plasmocytes (*P* = 0.002) in comparison with cluster 1 and more lymphoid aggregates than cluster 3 (*P* = 0.02); (iii) cluster 3, defined as neutrophil-rich infiltrate was primarily characterized by a high number of neutrophils as compared with the other 2 clusters (*P* < 0.0001 for both clusters) (Figure 1a–h, Supplementary Table S3). Of note, the immune infiltrate in glomeruli was minimal and showed no significant difference between the clusters (not shown), aligning with the pathological diagnosis of ICI-AIN.

### Clinical and Biological Characteristics Across the 3 Clusters at Baseline and at ICI-AIN Diagnosis

The clinical and biological characteristics of the 3 clusters at baseline and at ICI-AIN diagnosis are presented in detail in Table 1. There were no statistically significant differences between the clusters in terms of age at the time of kidney biopsy (*P* = 0.5), baseline estimated glomerular filtration rate (*P* = 0.7), or proton pump inhibitor use (*P* = 0.6). Nonsmall cell lung carcinoma was the most common cancer type across all clusters (9 [50%], 5 [33%], and 11 [69%] in clusters 1, 2, and 3, respectively; *P* = 0.4). Anti-PD1 therapy was the most frequently used ICI in all 3 groups (17 [94%], 15 [100%], and 15 [94%] in clusters 1, 2, and 3, respectively; *P* > 0.9) (Table 1 and Supplementary Table S4). However, baseline NLR was significantly higher in patients from cluster 3 (5.7 [3.4–8.5]) in comparison with cluster 1 (3.4 [2.7–4.8]) and cluster 2 (2.9 [2.1–4.2]), (*P* = 0.005) (Table 1).

**Figure 2:**
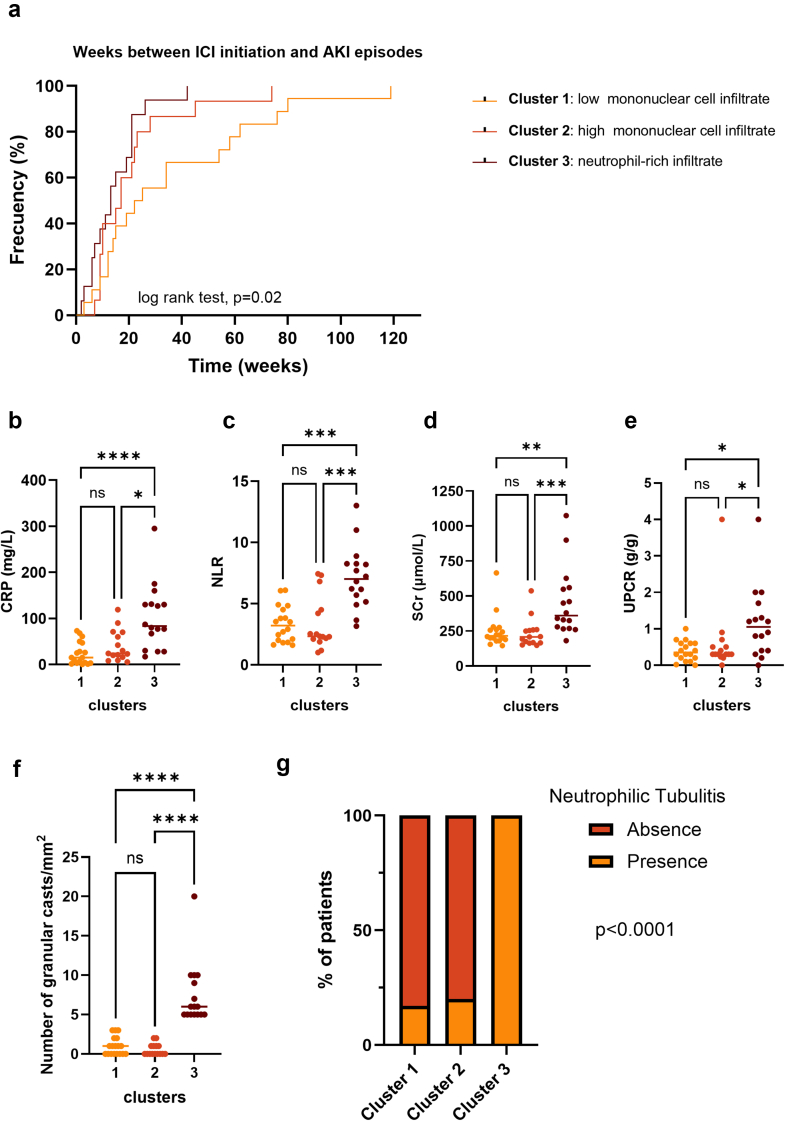
Description of ICI-induced acute interstitial nephritis events. (a) Cumulative incidence curves representing the time from ICI initiation to the onset of AKI in the 3 clusters (log-rank test). (b) Comparison of peak CRP levels during ICI-induced interstitial nephritis events among the 3 clusters. (c) Comparison of NLR levels among the 3 clusters. (d) Comparison of peak SCr levels among the 3 clusters. (e) Comparison of peak UPCR levels among the 3 clusters. (f) Quantification of granular casts per mm^2^ in each cluster. (g) Histogram of the proportion of the detected neutrophilic tubulitis in each cluster. ∗*P* ≤ 0.05; ∗∗*P* < 0.01; ∗∗∗*P* < 0.001; ∗∗∗∗*P <* 0.0001. Log-rank test for cumulative incidence curves and Kruskal–Wallis test with Dunn’s multiple comparison test for quantitative variables and Fisher exact test for qualitative variables when comparing 3 groups (b–e). AKI, acute kidney injury; CRP, C-reactive protein; ICI, immune checkpoint inhibitor; NLR, neutrophil-to-lymphocyte ratio; ns, not significant; SCr, serum creatinine; UPCR, urine protein-to-creatinine ratio.

Regarding features at ICI-AIN diagnosis, the median delay between ICI initiation and AKI in the entire cohort was 14 (9–24) weeks. At 14 weeks, 33% of patients in cluster 1, 47% in cluster 2, and 57% in cluster 3 had developed AKI (log rank test, *P* = 0.02) (Figure 2a). The proportion of extrarenal irAEs was comparable in all 3 groups (5 [28%], 3 [20%], and 4 [25%] in clusters 1, 2, and 3, respectively; *P* = 0.9) (Table 1). However, the neutrophil-rich cluster 3 interestingly showed more biological evidence of systemic inflammation than the other 2 clusters. We identified significantly higher C-reactive protein levels at ICI-AIN diagnosis (84 [39–131] mg/l in cluster 3 vs. 15 [3–48] mg/l and 24 [16–70] mg/l in clusters 1 and 2, respectively; *P* = 0.0002), as well as a higher NLR in blood (7 [5.3–8.3] in cluster 3 vs. 3.2 [1.9–4.6] and 2.3 [2.1–4.5] in clusters 1 and 2, respectively; *P* < 0.0001) (Figure 2b and c and Table 1). Importantly, patients in cluster 3 had higher levels of peak SCr than the other 2 clusters (360 [271–557] μmol/l in cluster 3 vs. 215 [187–263] μmol/l and 208 [165–258] μmol/l in clusters 1 and 2, respectively; *P* = 0.0001) and displayed higher levels of urinary protein-to-creatinine ratio than those in cluster 1 (1 [0.4–1.6] g/g vs. 0.3 [0.2–0.6]g/g and 0.3 [0.2–0.5] g/g in clusters 1 and 2, respectively; *P* = 0.02) (Figure 2d and e). Of note, 3 patients required hemodialysis at diagnosis (2 patients from cluster 3 and 1 from cluster 1). Overall, these data show that the neutrophil-rich cluster 3 exhibits distinct biological features at ICI-AIN diagnosis, including more severe renal failure and a stronger biological inflammatory response compared with the other 2 groups.

### Standard Histological Analysis of the 3 Clusters

To identify potential morphological features specific to each of the 3 clusters, kidney biopsies from all patients were blindly reassessed by 2 kidney pathologists (PI and MR). We performed a semiquantitative assessment of the observed kidney lesions. Kidney pathological findings are summarized in Table 2 and illustrated Supplementary Figure S3. Importantly, kidney biopsies were comparable between the 3 clusters in terms of biopsy size (i.e., total number of glomeruli, *P* = 0.2) and chronic changes (i.e., number of sclerotic glomeruli, *P* = 0.6, and interstitial fibrosis and tubular atrophy, *P* = 0.7). Careful morphological analysis of kidney biopsies confirmed that patients in cluster 1 exhibited less interstitial immune infiltrate, particularly with significantly lower inflammation in interstitial fibrosis and tubular atrophy (*P* = 0.0003) and less peritubular capillaritis (*P* = 0.0001) compared with cluster 3 (Table 2). These differences did not reach statistical significance when compared with cluster 2. Patients in cluster 3 exhibited significantly more granular casts, with a minimum of 5 granular casts/mm^2^ observed in this group, compared with a maximum of 3 granular casts/mm^2^ in clusters 1 and 2 (*P* < 0.0001). In addition, neutrophilic tubulitis was present in 100% of patients in cluster 3, whereas it was observed in only 11% and 13% of patients in clusters 1 and 2, respectively (*P* < 0.0001) (Figure 2f and g). Furthermore, eosinophilic infiltration was significantly higher in cluster 3 compared with cluster 2 (*P* = 0.01). These observations underscore the morphological specificity of the neutrophil-rich cluster 3, which is distinguished by a readily identifiable interstitial infiltrate of neutrophils, accompanied by acute tubular inflammatory lesions such as neutrophilic tubulitis and casts. To qualitatively validate our multiplex immunofluorescence-based data and assess its suitability for routine clinical pathology techniques, we performed anti-CD3 (T lymphocyte), anti-CD20 (B lymphocyte), anti-CD68 (macrophage) and anti-CD15 neutrophil immunohistochemistry staining. We were able to confirm the neutrophil-rich nature of cluster 3, with its numerous neutrophilic tubulitis and casts, the dense mononuclear (lymphocytic and macrophagic) infiltrate of cluster 2, and the visually lower immune infiltrate of cluster 1 (Supplementary Figure S3).

**Table 2:** Renal histology across the 3 clusters

	Whole Cohort *N* = 49	Cluster 1 *n* = 18	Cluster 2 *n* = 15	Cluster 3 *n* = 16	*P*-value^b^
Number of glomeruli, median (IQR)	15 (10-23)	17 (11-32)	19 (10-21)	13 (10-18)	0.2
Number of sclerotic glomeruli, median (IQR)	1 (0-3)	2 (1-3)	1 (0-4)	1 (0-3)	0.6
ATN, median (IQR)	2 (1-2)	1 (1-2)	1.5 (1-2)	2 (1-3)	0.07
Interstitial edema, median (IQR)	2 (1-2)	1 (1-2)	2 (1-2)	2 (1-3)	0.05
Interstitial Inflammation, median (IQR)	2 (1-3)	1.5 (1-2.2)	2 (2-2)	2 (1.3-3)	0.1
Eosinophil ratio^a^	0.3 (0-1.5)	0.4 (0-0.9)	0.3 (0-0.5)	2 (0.2-2.3)	0.01
Tubulitis, median (IQR)	3 (2-3)	3 (2-3)	3 (2-3)	3 (3-3)	0.09
Presence of Neutrophilic tubulitis, *n* (%)	22 (45)	2 (11)	2 (13)	18 (100)	< 0.0001
Granular casts, median (IQR)	1 (0-5)	1 (0-2)	0 (0-1)	6 (5-10)	< 0.0001
IF/TA, median (IQR)	2 (1-2)	2 (1-2)	1 (1-3)	2 (1-3)	0.7
i-IF/TA, median (IQR)	2 (1-3)	1 (1-2)	2 (1-3)	3 (2-3)	0.0009
ptc, median (IQR)	2 (1-2)	1 (0-2)	2 (1-2)	2 (2-3)	0.0002
cv, median (IQR)	1 (1-2)	1 (0.5-1.5)	2 (0-2)	1 (1-2)	0.6
ah, median (IQR)	1 (0-2)	0.5 (0-1.3)	2 (0-2)	1 (0-2)	0.2

ah, arteriolar hyalinosis; ATN, acute tubular necrosis; cv, arteriosclerosis; IF/TA, interstitial fibrosis and tubular atrophy; i-IF/TA, inflammation in areas of fibrosis; IQR, interquartile range; ptc, peritubular capillaritis.

Banff scores (0: no significant lesion; 1: mild; 2: moderate; 3: severe).

a The eosinophil ratio was calculated by dividing the number of eosinophils counted at 20× magnification on the frozen kidney biopsy by the number of fields analyzed at 20× magnification.

b The *P*-value corresponds to the comparison between patients in the 3 clusters (not the entire cohort), using the Kruskal-Wallis test for quantitative variables and Fisher exact test for qualitative variables.

### Neutrophil-Rich Infiltration Within the Kidneys is Unlikely to be Infection-Driven

Considering that infectious diseases are a well-known cause of AIN, particularly for neutrophil-rich forms (cluster 3), which closely resemble the histological lesions seen in acute pyelonephritis, we thoroughly reviewed the urine cytology and culture tests of all patients. All patients had negative urine cultures, including the 13 patients in cluster 3. Moreover, we conducted metagenomic next-generation sequencing directly on frozen kidney biopsy samples from 7 of the 13 neutrophil-rich cluster patients (54%). No pathogen sequences were detected in any of the kidney biopsies.

### Kidney Recovery After Treatment

ICI and proton pump inhibitors were withdrawn in all patients, who subsequently received corticosteroid treatment over a period of 6 weeks. The steroid regimens were not significantly different among the 3 groups (individual data in Supplementary Table S4). At 3 months postinitiation, patients in cluster 2 (high mononuclear cell infiltrate) demonstrated a superior response compared with those in clusters 1 and 3, as evidenced by significantly higher rates of complete recovery (11 [73%] in cluster 2 vs. 5 [28%] and 2 [13%] in clusters 1 and 3, respectively; *P* = 0.001) and lower SCr levels (90 [81–117] μmol/l in cluster 2 vs. 140 [104–170] μmol/l and 177 [138–213] μmol/l in clusters 1 and 3, respectively; *P* < 0.0001).

**Figure 3:**
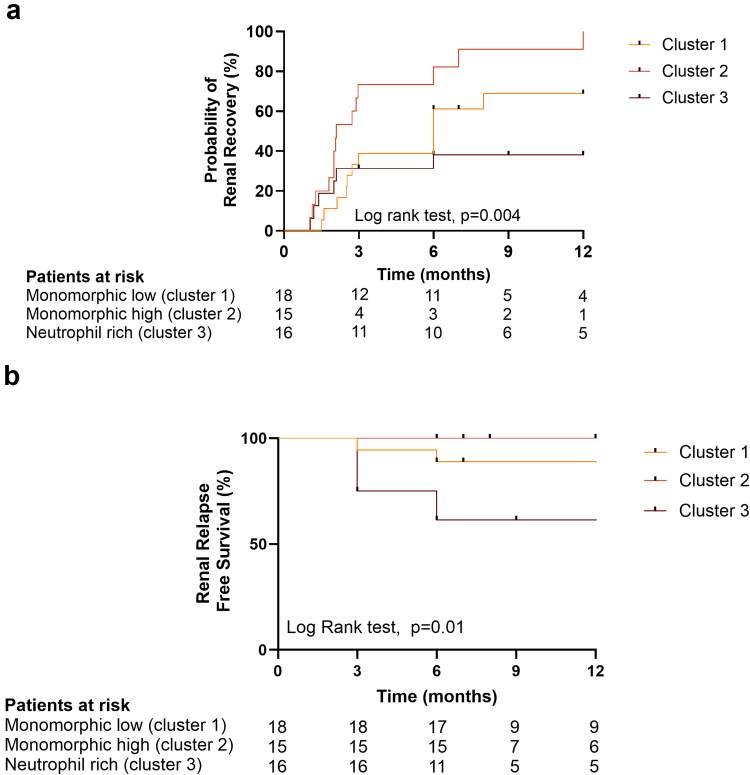
Renal outcomes across the 3 clusters following steroid treatment. (a) Kaplan-Meier curves of renal response to steroids across the 3 clusters in the year following steroid initiation. (b) Kaplan-Meier curves of relapse-free renal survival in the year following steroid initiation across the 3 clusters. Renal response was defined as at least a partial response (serum creatinine returning to < 31 μmol/l or increase in serum creatinine > 31 μmol/l but remaining < 2× the baseline value, or as the discontinuation of renal replacement therapy), regardless of SCr value. (a) ∗*P* ≤ 0.05; ∗∗*P* < 0.01; ∗∗∗*P* < 0.001; ∗∗∗∗*P* < 0.0001. Mann–Whitney test for 2-group comparisons. (b and c) log rank test. SCr, serum creatinine.

Notably, 1-year renal response rates (defined as at least a partial recovery) differed significantly across clusters: 93% in cluster 2, 67% in cluster 1, and 38% in cluster 3 (log-rank test, *P* = 0.004, Figure 3a). Dialysis was successfully discontinued in all 3 patients who initially required renal replacement therapy, although 1 patient in cluster 3 progressed to end-stage kidney disease after 24 months of follow-up. Overall, these data suggest that a high mononuclear cell infiltrate (cluster 2) may be associated with greater steroid sensitivity, whereas neutrophil-rich (cluster 3) or low mononuclear cell infiltrate (cluster 1) appear to respond less favorably to steroids.

Importantly, 1-year relapse rates following ICI-AIN diagnosis differed significantly across clusters, with the highest probability of recurrence in neutrophil-rich cluster 3 (38%), compared with 11% in cluster 1 and 0% in cluster 2 (log-rank test, *P* = 0.01) (Figure 3b**)**. All relapses occurred between 1 and 3 months after discontinuation of corticosteroids, without reinitiation of ICIs. Three patients underwent repeat biopsies during follow-up due to incomplete recovery (*n* = 1, cluster 1) or suspected relapse (*n* = 2, cluster 3). The repeat biopsy from the cluster 1 patient revealed no active inflammation but showed worsening fibrosis. In contrast, both cluster 3 patients exhibited a recurrence of active, neutrophil-rich inflammation, despite the discontinuation of ICI therapy (as illustrated for 1 patient in Supplementary Figure S4). Consistently, in cluster 3, NLR increased at the time of relapse compared with patients who did not relapse (Supplementary Figure S5). Finally, ICIs were rechallenged in 4 patients from clusters 1 (*n* = 2) and 2 (*n* = 2) under 10 mg of prednisone, with no relapse of AKI.

### Implication of C5a/C5aR1 Axis in Neutrophil-Rich ICI-AIN

**Figure 4:**
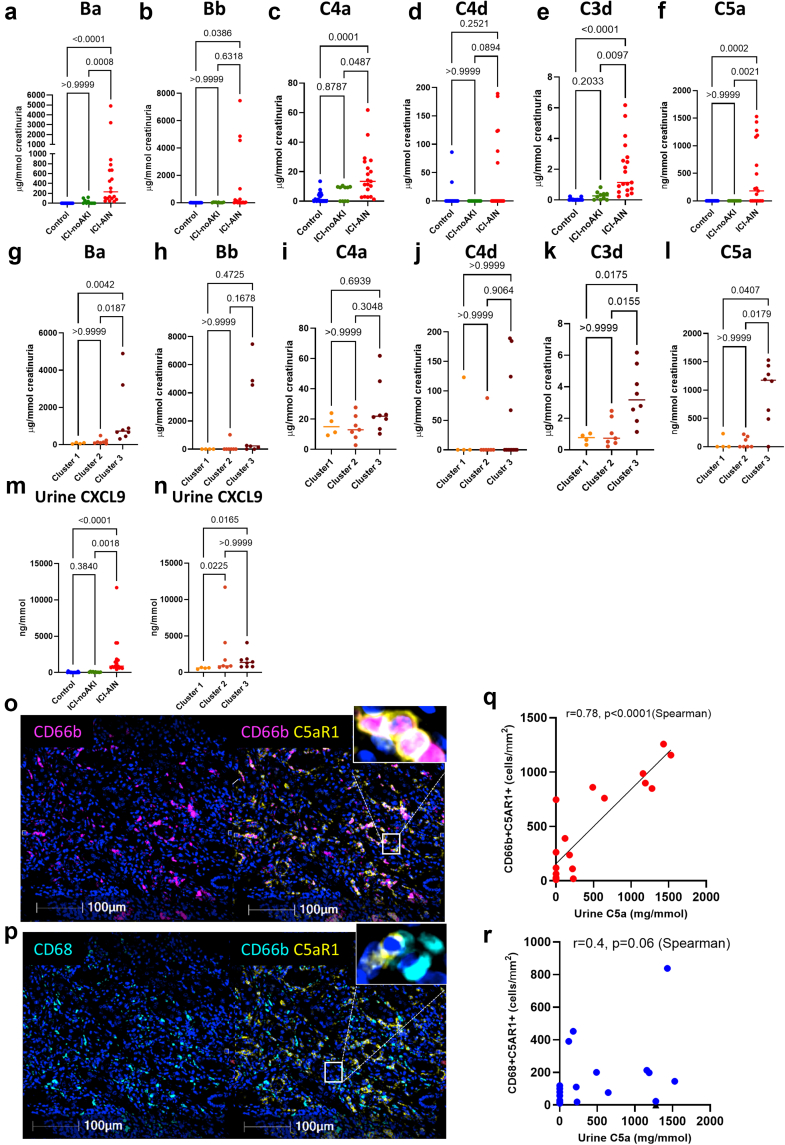
Complement activation in ICI-AIN drives C5a generation and recruits C5aR1+ neutrophils in neutrophil-rich ICI-AIN. (a–f) Comparison of complement activation markers in the urine of healthy donors (control, blue) versus ICI-noAKI (green) and patients with ICI-AIN (red): (a) Ba, (b) Bb, (c) C4a, (d) C4d, (e) C3d, and (f) C5a. (g–l**)** Complement activation markers in patients with ICI-AIN stratified by cluster 1 to 3: (g) Ba, (h) Bb, (i) C4a, (j) C4d, (k) C3d, and (l) C5a. (m–n) Urine CXCL9 levels: (m) healthy donors (control, blue) versus ICI-noAKI (green) and patients with ICI-AIN (red); (n) patients with ICI-AIN from clusters 1, 2 and 3. (o and p) Representative images of immune cells expressing C5aR1: (o) neutrophils (CD66b+, magenta) expressing C5aR1 (yellow); (p) macrophages (CD68+, cyan) expressing C5aR1 (yellow). (q and r) Correlation between urine C5a levels and C5aR1+ immune cells: (q) C5aR1+ neutrophils; (r) C5aR1+ macrophages. ∗*P* ≤ 0.05; ∗∗*P* < 0.01; ∗∗∗*P* < 0.001; ∗∗∗∗*P* < 0.0001. Mann–Whitney test for 2-group comparisons. Spearman correlation for k and l. ICI, immune checkpoint inhibitor; ICI-AIN, ICI-induced acute interstitial nephritis; ICI-noAKI, patients treated with ICI without AKI.

Given the critical role of the complement system in leucocyte recruitment and activation (especially neutrophils) in several tissues, we assessed the presence of complement-activation fragments, including C5a, in urine samples from 19 patients in the cohort: 4 from cluster 1, 7 from cluster 2, and 8 from cluster 3. Compared with healthy donors (*n* = 17) and controls of ICI-noAKI (*n* = 10), patients with ICI-AIN (regardless of neutrophil infiltration) exhibited significantly elevated levels of complement fragments, particularly Ba (231 [97–670] μg/mmol vs. undetectable in healthy donors and 0 [0–67] μg/mmol in ICI-noAKI, *P ≤* 0.0001), C4a (13 [3–27] μg/mmol vs. 1 [0–5] μg/mmol in healthy donors and 13 [0–27] μg/mmol in ICI-noAKI, *P* = 0.0002), C3d (1.1 [0.6–2.8] μg/mmol vs. 0.01 [0–0.02] μg/mmol in healthy donors and 0.3 (0.01-0.4) in ICI-noAKI, *P* < 0.0001), and C5a (180 [0–1158] μg/mmol vs. undetectable in healthy donors and ICI-noAKI, *P* < 0.0001, Figure 4a–f). C3a and sC5b-9 were not detected except in 2 patients from cluster 3. Among patients with ICI-AIN, Ba, C3d, and C5a activation fragments were significantly higher in patients from cluster 3 than in patients from clusters 1 and 2 (*P* = 0.0001, *P* = 0.0008, and *P* < 0.0001, respectively for Ba, C3d, and C5a, Figure 4g–l**)**. Conversely, urine CXCL9, an IFN-γ–induced chemokine involved in lymphocyte chemotaxis,^13^^,^^26^ was significantly elevated in patients with ICI-AIN compared with healthy donors and ICI-noAKI (Figure 4m**)**. Likewise, urine CXCL9 levels were significantly higher in patients in clusters 3 and 2 than in those in cluster 1 (*P* = 0.02 for both) but showed no significant difference between clusters 2 and 3 patients (*P* > 0.9, Figure 4n).

To further explore the relationship between urinary C5a levels and infiltration by C5aR1+ immune cells in kidney biopsies, we added C5aR1 staining to our multiplex immunofluorescence panel used for kidney biopsies analysis. As expected, C5aR1 was predominantly expressed by neutrophils and macrophages (Figure 4o and p). Interestingly, a significant correlation was observed between urinary C5a levels and degree of tissular infiltration by C5aR1-positive neutrophils (CD66b+ and C5aR1+) (Spearman correlation, rho = 0.78, *P* < 0.0001), whereas the correlation did not reach significance for macrophages (Spearman correlation, CD68+ C5aR1+, rho = 0.4, *P* = 0.06) **(**Figure 4k and l), suggesting a link between complement activation and neutrophil activation and chemotaxis.

## Discussion

Understanding the mechanisms underlying ICI-AIN is essential, because this toxicity can cause irreversible renal injury and influence cancer management. Using multiplex immunofluorescence and detailed pathological assessment, we identified distinct immune infiltration patterns associated with specific clinico-biological profiles and treatment responses. A key finding was the identification of a neutrophil-rich subtype associated with more severe presentations, reduced steroid responsiveness, and a higher risk of relapse

Unsupervised clustering identified 3 ICI-AIN clusters based on immune phenotyping: Clusters 1 and 2 had predominant mononuclear cell infiltrates, with low and high immune cell abundance, respectively, but minimal neutrophilic infiltration. Cluster 3 was distinct, dominated by neutrophils, resembling acute bacterial pyelonephritis. Standard Banff scoring did not fully capture these compositional differences, underscoring the added value of quantitative, cell-type–resolved imaging for refining histologic assessment of ICI-AIN.

Clinically, cluster 3 showed the highest systemic inflammation, with elevated C-reactive protein; neutrophil counts; and NLR, a biomarker previously linked to increased irAE risk.^17^, ^18^, ^19^ Notably, NLR was already elevated at baseline in patients from cluster 3 and further increased during relapses, suggesting that systemic neutrophil activation may parallel, or even anticipate, AKI. Although preliminary, these observations support the need for larger studies to determine whether NLR could help identify patients predisposed to developing a neutrophilic and potentially more severe form of ICI-AIN. Similar infiltrates are seen in other irAEs, like upper gastritis, where infections such as *Helicobacter pylori* must be excluded.^27^^,^^28^ Importantly, in our study, infections were rigorously excluded through negative urine cultures and metatranscriptomic analyses.

Steroid responses differed across clusters. Cluster 1 showed limited improvement, likely due to sparse inflammatory infiltrates that may have progressed to fibrosis, as supported by a rebiopsy^8^^,^^29^; whereas cluster 2 responded well, with all patients achieving at least partial remission. Cluster 3, though showing improvement with corticosteroids, had a lower overall renal response and more frequent relapses. Rebiopsies revealed persistent neutrophil-rich patterns, suggesting that autoimmune phenomena may contribute to persistent or recurring inflammation after ICI discontinuation. Cluster 3 represents a severe subgroup that may need prolonged or intensified corticosteroid treatment and/or adjunctive therapies targeting this subgroup's pathophysiology, highlighting the need for subtype-specific therapeutic strategies in ICI-AIN.

Mechanistically, complement activation may contribute to this neutrophil-rich form. Urinary complementomic profiling^23^^,^^24^ showed increased C5a levels in ICI-AIN, with the highest concentrations in cluster 3. Urinary C5a correlated with C5aR1-positive neutrophils, supporting a link between complement activation and neutrophil recruitment. These observations raise the possibility that C5a/C5aR1 signaling contributes to disease severity and could represent a therapeutic target, particularly given recent approval of C5aR1 inhibition for antineutrophil cytoplasmic autoantibody vasculitis^30^ and preclinical data suggesting synergy with ICIs.^31^, ^32^, ^33^, ^34^

The emergence of distinct immune phenotypes in ICI-AIN may reflect a combination of host immune predisposition, the timing and intensity of immune activation, local renal microenvironmental cues, and concomitant medications. These factors may determine whether the infiltrate is predominantly mononuclear, mild, or neutrophil-rich, with important implications for severity, steroid responsiveness, and relapse risk. Future studies incorporating transcriptomic and mechanistic analyses are needed to elucidate the pathways underlying these divergent immune responses.

This study has limitations. Its retrospective design and small cohort reduce statistical power, although the differences observed between clusters support their biological relevance. The limited number of events precluded multivariable analyses and the short follow-up restricts conclusions on long-term renal outcomes. In addition, our immunofluorescence panel included a limited set of immune markers, notably lacking dendritic cell markers, which may have constrained the characterization of tertiary lymphoid structure–like aggregates; their degree of maturity, rather than number, may be clinically relevant. Of note, eosinophils were also increased in Cluster 3, suggesting possible drug-related hypersensitivity, a point that future studies incorporating eosinophil-specific markers should explore. Hyperplex imaging will enable more comprehensive profiling of complement pathways and immune phenotypes. Finally, whether complement activation fragments represent a specific biomarker of neutrophil-rich ICI-AIN remains to be determined in a larger cohort, and their association with tubular injury warrants further investigation.

In conclusion, we identified distinct ICI-AIN subtypes with varying presentations and outcomes, including a neutrophil-rich cluster potentially linked to complement activation. Prospective studies with larger cohorts, multimodal phenotyping, and longer follow-up are needed to confirm the pathophysiological and clinical significance of immune infiltrates and complement activation in ICI-AIN. Such studies may lead to new diagnostic and therapeutic strategies for personalized healthcare in ICI-treated patients.

## Disclosure

All the authors declared no competing interests.

## Supplementary Material

Supplementary File (PDF) **Supplementary Methods. Figure S1.** Study flow chart. **Figure S2.** Representative images of (A) lymphoid aggregates and (B) diffuse lymphocyte infiltration in patients with ICI-AIN. **Figure S3.** Morphological and immunochemistry analysis of ICI-AIN kidney biopsies. **Figure S4.** Example of AIN recurrence in a patient from cluster 3, occurring 6 months after immunotherapy discontinuation and corticosteroid cessation. **Figure S5.** Evolution of the neutrophil-to-lymphocyte ratio (NLR) over time in cluster 3 according to relapse status. **Table S1.** Multiplex immunofluorescence protocol. **Table S2.** Control patients treated with immune checkpoint inhibitors without AKI, used for urinary complement activation fragment analysis. **Table S3.** Immune cell density across the 3 clusters. **Table S4.** Individual data on corticosteroid treatment and treatment response. STROBE Checklist.

## Footnotes

Supplementary File (PDF)

**Supplementary Methods.**
**Figure S1.** Study flow chart. **Figure S2.** Representative images of (A) lymphoid aggregates and (B) diffuse lymphocyte infiltration in patients with ICI-AIN. **Figure S3.** Morphological and immunochemistry analysis of ICI-AIN kidney biopsies. **Figure S4.** Example of AIN recurrence in a patient from cluster 3, occurring 6 months after immunotherapy discontinuation and corticosteroid cessation. **Figure S5.** Evolution of the neutrophil-to-lymphocyte ratio (NLR) over time in cluster 3 according to relapse status. **Table S1.** Multiplex immunofluorescence protocol. **Table S2.** Control patients treated with immune checkpoint inhibitors without AKI, used for urinary complement activation fragment analysis. **Table S3.** Immune cell density across the 3 clusters. **Table S4.** Individual data on corticosteroid treatment and treatment response. STROBE Checklist.
