# Supplementary material for: Immune Profiling Identifies High-Risk Neutrophil-Rich Subtype in Checkpoint Inhibitor Nephritis
Source: Kidney Int Rep. 2026 Jan 6;11(3):103766. doi: 10.1016/j.ekir.2025.103766 (PMC12918167; doi:10.1016/j.ekir.2025.103766)
Supplement: Supplementary File (PDF) — Supplementary Methods. Figure S1. Study flow chart. Figure S2. Representative images of (A) lymphoid aggregates and (B) diffuse lymphocyte infiltration in patients with ICI-AIN. Figure S3. Morphological and immunochemistry analysis of ICI-AIN kidney biopsies. Figure S4. Example of AIN recurrence in a patient from cluster 3, occurring 6 months after immunotherapy discontinuation and corticosteroid cessation. Figure S5. Evolution of the neutrophil-to-lymphocyte ratio (NLR) over time in cluster 3 according to relapse status. Table S1. Multiplex immunofluorescence protocol. Table S2. Control patients treated with immune checkpoint inhibitors without AKI, used for urinary complement activation fragment analysis. Table S3. Immune cell density across the 3 clusters. Table S4. Individual data on corticosteroid treatment and treatment response. STROBE Checklist. [file mmc1.pdf]

Supplementary material

Supplemental Methods

Metatranscriptomics

Total RNA was extracted from kidney tissue using the ELITe InGenius instrument (ELITechGroup) after an initial step of tissue disruption with Bertin beads and a Precellys homogenizer (Bertin Technologies). A DNAse treatment was applied with the TURBO DNase (Invitrogen). The positive internal control ZymoBIOMICS Spike-in Control (Zymo research) was added to all samples prior to extraction. NGS libraries were constructed with the SMARTer Stranded Total RNA-Seq Kit - Pico Input Mammalian Kit v.3 (Takara Bio) and sequenced in 1x150bp on a NextSeq500 instrument (Illumina) using a High Output flow cell. NGS libraries were sequenced with a minimum of 50 million reads. The search for pathogens was performed with bioinformatics pipelines using a protein-based approach for the detectio of viruses, while a k-mer (Kraken-2, Centrifuge) and marker-based (Metaphlan 2) approach were used to detect other pathogens (bacteria, fungi, eukaryotes). Details of the protocols, kits, quality control, positive controls and databases used have been described previously[25]

Supplemental Tables:

- Supplemental Table 1: Multiplex Immunofluorescence protocol.
- Supplemental Table 2*.* Control patients treated with immune checkpoint inhibitors without AKI, used for urinary complement activation fragment analysis.
- Supplemental Table 3: Individual Data on Corticosteroid Treatment and Treatment Response
- Supplemental Table 4: Immune cell density across the three clusters.

Supplemental Figures:

- Supplemental Figure 1: Study Flow chart.
- Supplemental Figure 2: Representative images of lymphoid aggregates (A) and diffuse lymphocyte infiltration (B) in ICI-AIN patients
- Supplemental Figure 3: Morphological and immunochemistry analysis of ICI-AIN kidney biopsies

- Supplemental Figure 4: Example of AIN recurrence in a patient from cluster 3, occurring six months after immunotherapy discontinuation and corticosteroid cessation.

- Supplemental Figure 5. Evolution of the neutrophil-to-lymphocyte ratio (NLR) over time in Cluster 3 according to relapse status**.**

- STROBE Checklist

Supplemental Table 1. Multiplex Immunofluorescence protocol.

Primary Antibody	Dilution	Secondary antibody	OPAL TSA Fluorophore
CD68 (MO814, Dako)	1:200	Anti Ms + Rb HRP (ARH1001EA)	480
CD66b (55723, BD)	1:200	Anti Ms + Rb HRP (ARH1001EA)	650
CD3 (M7254, DAKO)	1:200	Anti Ms + Rb HRP (ARH1001EA)	620
CD20 (MO75501, DAKO)	1:400	Anti Ms + Rb HRP (ARH1001EA)	690
MUM1 (7259, DAKO)	1:100	Anti Ms + Rb HRP (ARH1001EA)	520
CD34 (M7165, DAKO)	1:200	Anti Ms + Rb HRP (ARH1001EA)	780
C5aR1, 8D6 (Santa Cruz)	1:200	Anti-rat (MP-7404, Vector Laboratories)	570

Abbreviations: Ms: mouse; Rb: Rabbit; HRP: horseradish peroxidase

Supplemental Table 2. *Control patients treated with immune checkpoint inhibitors without acute kidney injury, used for urinary complement activation fragment analysis.*

	Age	Sex	Cancer	Treatment	Blood Creatinin	CRP	NLR
1	68	Male	NSCLC	Carboplatin, Pemetrexed, Pembrolizumab	80	1,5	3,85483871
2	60	Female	NSCLC	Carboplatin, Pemetrexed, Pembrolizumab	51	11,3	2,27631579
3	69	Female	NSCLC	Carboplatin, Taxol, Pembrolizumab	51	8	2,60833333
4	69	Male	NSCLC	Carboplatin, Pemetrexed, Cemiplibab	97,2	2	2,64705882
5	59	Male	NSCLC	Pembrolizumab	74	33	5,01315789
6	66	Male	NSCLC	Durvalumab	90	1,9	1,54545455
7	70	Male	NSCLC	Carboplatin, Pemetrexed, Pembrolizumab	111	7	1,81497797
8	58	Male	NSCLC	Durvalumab	73	4	2,08658009
9	51	Male	NSCLC	Carboplatin, Pemetrexed, Pembrolizumab	78	9,4	0,92976589
10	71	Female	NSCLC	Carboplatin, Pemetrexed, Pembrolizumab	80	6	2,28996283

Supplemental Table 3: Immune cell density across the three clusters.
IQR: interquartile range. *The p-value corresponds to the comparison between the three clusters using Kruskall Wallis test

Cell Marker	Whole Cohort N=49	Cluster 1 N=18	Cluster 2 N=15	Cluster 3 N=16	p-Value*
T lymphocytes (CD3+), cells/mm^2, median [IQR]	277 [184-453]	240 [181-307]	513 [340-689]	226 [146-286]	0.0002
B lymphocytes (CD20+), cells/mm^2, median [IQR]	331 [136-474]	207 [96-303]	554 [370-792]	296 [117-460]	0.0001
Plasmocytes (MUM1+), cells/mm^2, median [IQR]	44 [18-69]	13 [7-37]	59 [36-82]	63 [39-81]	0.0002
Macrophages (CD68+), cells/mm^2, median [IQR]	175 [41-400]	40 [26-68]	329[209-430]	257 [115-399]	<0.0001
Neutrophils (CD66b+), cells/mm^2, median [IQR]	88 [22-1042]	42 [12-91]	51 [12-98]	1252 [1023-1457]	<0.001
Number of Lymphoid agregates, absolute number per biopsy, median [IQR]	2[1-3]	2 [1-2]	3 [1-5]	2 [0-1]	0.03

Patient	Cluster	Name of ICI	Corticosteroid regimen	Renal Recovery	Relapse (0=No ; 1= Yes)
1	1	Pembrolizumab	MP 500 mg × 3 IV boluses, then 1 mg/kg/d prednisone	complete	0
2	1	Avelumab	0.5 mg/kg prednisone	no recovery	0
3	1	Nivolumab	0,5mg/kg prednisone	no recovery	1
4	1	Nivolumab	0.5 mg/kg prednisone	complete	0
5	2	Nivolumab	0.5 mg/kg prednisone	complete	0
6	3	Pembrolizumab	0.5 mg/kg prednisone	partial	1
7	3	Pembrolizumab	1 mg/kg prednisone	partial	0
8	3	Pembrolizumab	0.5 mg/kg prednisone	no recovery	0
9	3	Pembrolizumab	1 mg/kg prednisone	no recovery	1
10	1	Nivolumab	1 mg/kg prednisone	partial	0
11	2	Pembrolizumab	0.5 mg/kg prednisone	partial	0
12	2	Nivolumab	0.5 mg/kg prednisone	complete	0
13	3	Pembrolizumab	0.5 mg/kg prednisone	partial	0
14	1	Nivolumab + Ipilumab	0.5 mg/kg prednisone	complete	0
15	3	Pembrolizumab	0.5mg/kg prednisone	no recovery	0
16	3	Nivolumab	2mg/kg prednisone	no recovery	0
17	1	Pembrolizumab	0.5 mg/kg prednisone	no recovery	0
18	3	Nivolumab	MP 500 mg × 3 IV boluses, then 1 mg/kg/d prednisone	no recovery	1
19	1	Pembrolizumab	1 mg/kg prednisone	complete	0
20	1	Pembrolizumab	0.5 mg/kg prednisone	complete	0
21	2	Pembrolizumab	1 mg/kg prednisone	complete	0
22	1	Pembrolizumab	1 mg/kg prednisone	no recovery	0
23	2	Pembrolizumab	1 mg/kg prednisone	complete	0
24	3	Nivolumab	1 mg/kg prednisone	complete	1
25	3	Nivolumab	1 mg/kg prednisone	no recovery	0
26	2	Nivolumab	1 mg/kg prednisone	complete	0
27	2	Nivolumab	0.5 mg/kg prednisone	complete	0
28	3	Atezolizumab	1 mg/kg prednisone	no recovery	1
29	2	Pembrolizumab	1 mg/kg prednisone	partial	0
30	1	Pembrolizumab	1 mg/kg prednisone	no recovery	0
31	2	Pembrolizumab	0.5 mg/kg prednisone	partial	0
32	2	Nivolumab	1 mg/kg prednisone	complete	0
33	2	Nivolumab + Ipilumab	1 mg/kg prednisone	partial	0
34	1	Pembrolizumab	0.5 mg/kg prednisone	partial	0
35	1	Pembrolizumab	MP 125 mg × 3 IV boluses, then 1 mg/kg/d prednisone	partial	0
36	1	Nivolumab	1 mg/kg prednisone	partial	0
37	3	Pembrolizumab	1 mg/kg prednisone	no recovery	0
38	2	Nivolumab	1 mg/kg prednisone	partial	0
39	3	Pembrolizumab	1 mg/kg prednisone	complete	0

40	1	Pembrolizumab	1 mg/kg prednisone	complete	0
41	1	Pembrolizumab	0.5 mg/kg prednisone	No recovery	1
42	1	Pembrolizumab	1 mg/kg prednisone	Partial	0
43	1	Nivolumab	0.5 mg/kg prednisone	Partial	0
44	2	Pemprolizumab	1 mg/kg prednisone	Complete	0
45	2	Pembrolizumab	1 mg/kg prednisone	Complete	0
46	2	Nivolumab	1 mg/kg prednisone	Partial	0
47	3	Pembrolizumab	1 mg/kg prednisone	No recovery	1
48	3	Nivolumab	1 mg/kg prednisone	No recovery	1
49	3	Nivolumab	1 mg/kg prednisone	Partial	0

Supplemental Table 4: Individual Data on Corticosteroid Treatment and Treatment Response

Supplemental Figure 1: Study Flow chart.

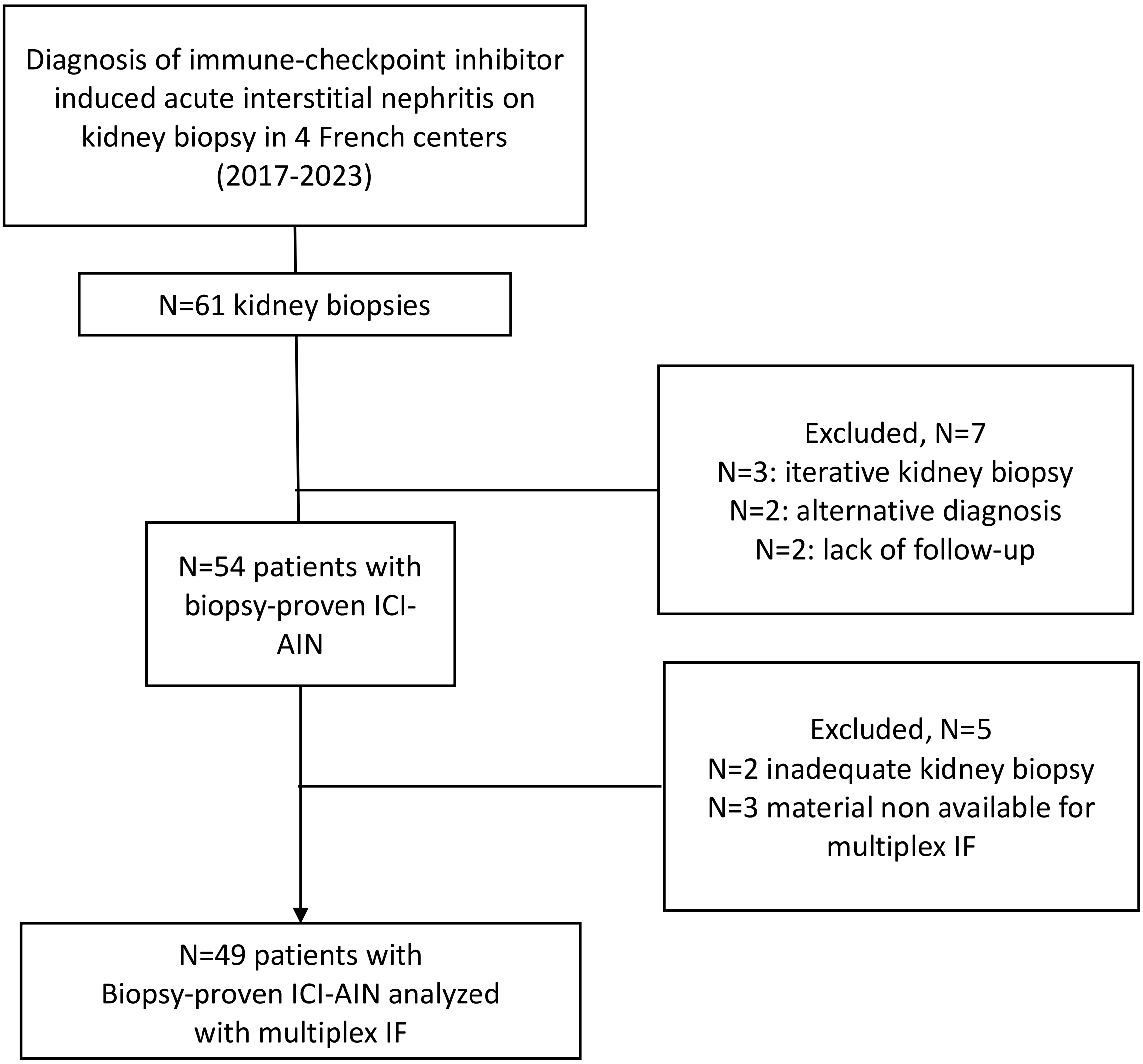

Abbreviations: ICI-AIN: Immune checkpoint inhibitor induced acute interstitial nephritis; IF: immunofluorescence.

Supplemental Figure 2: Representative images of lymphoid aggregates (A) and diffuse lymphocyte infiltration (B) in ICI-AIN patients.

A)

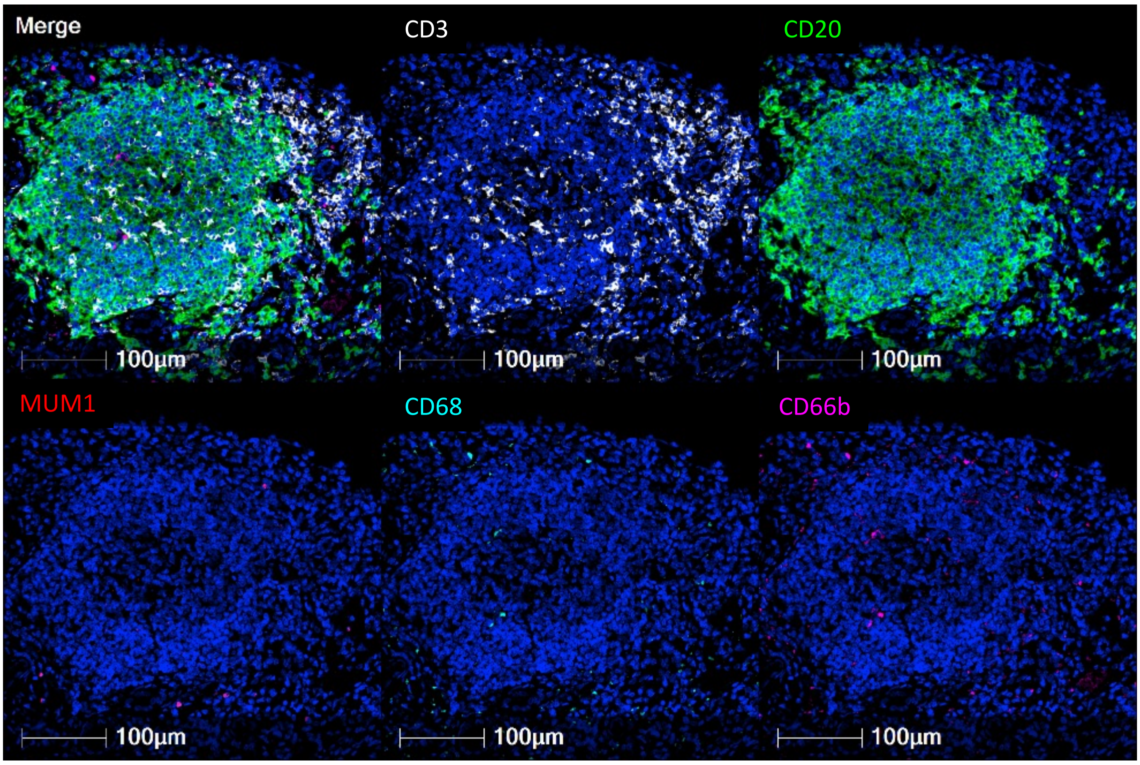

B)

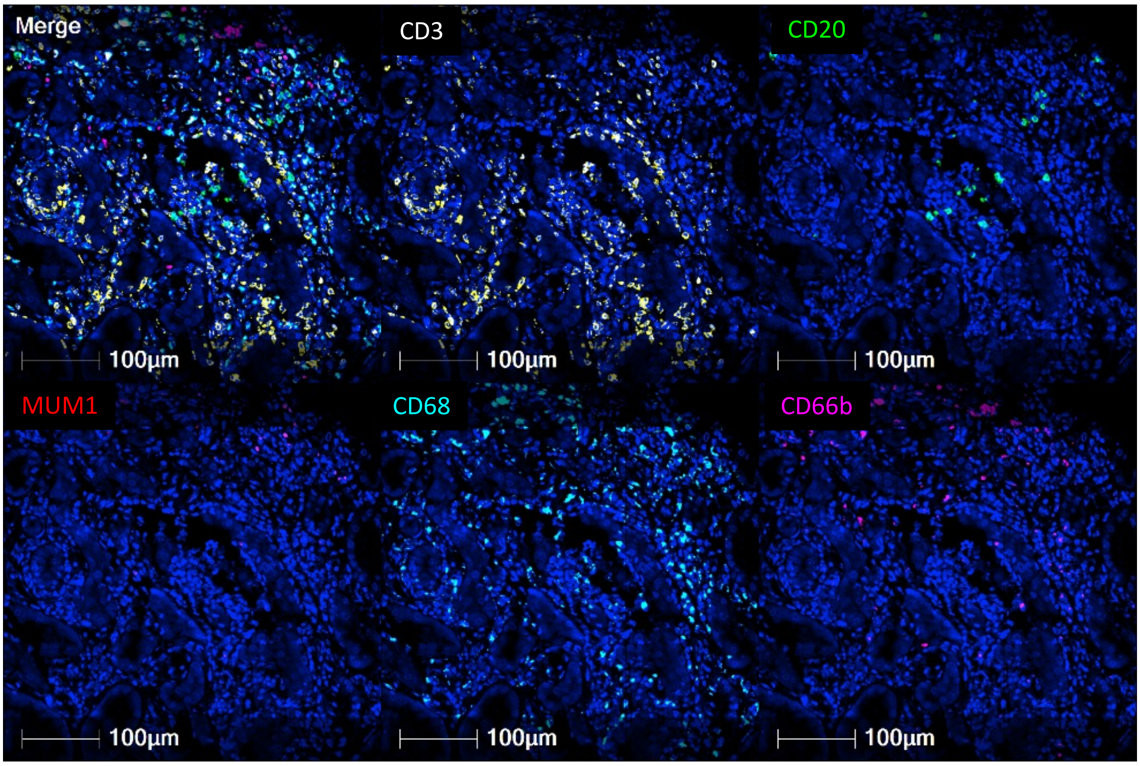

Original magnification 20X. Color codes are: CD3 (T cells, white), CD20 (B cells, green), MUM1 (plasmocytes, red), CD68 (macrophages, cyan) and CD66b (granulocytes, magenta). Nuclei are stained with DAPI (blue). Original Magnification x20. Scale bar 100µm

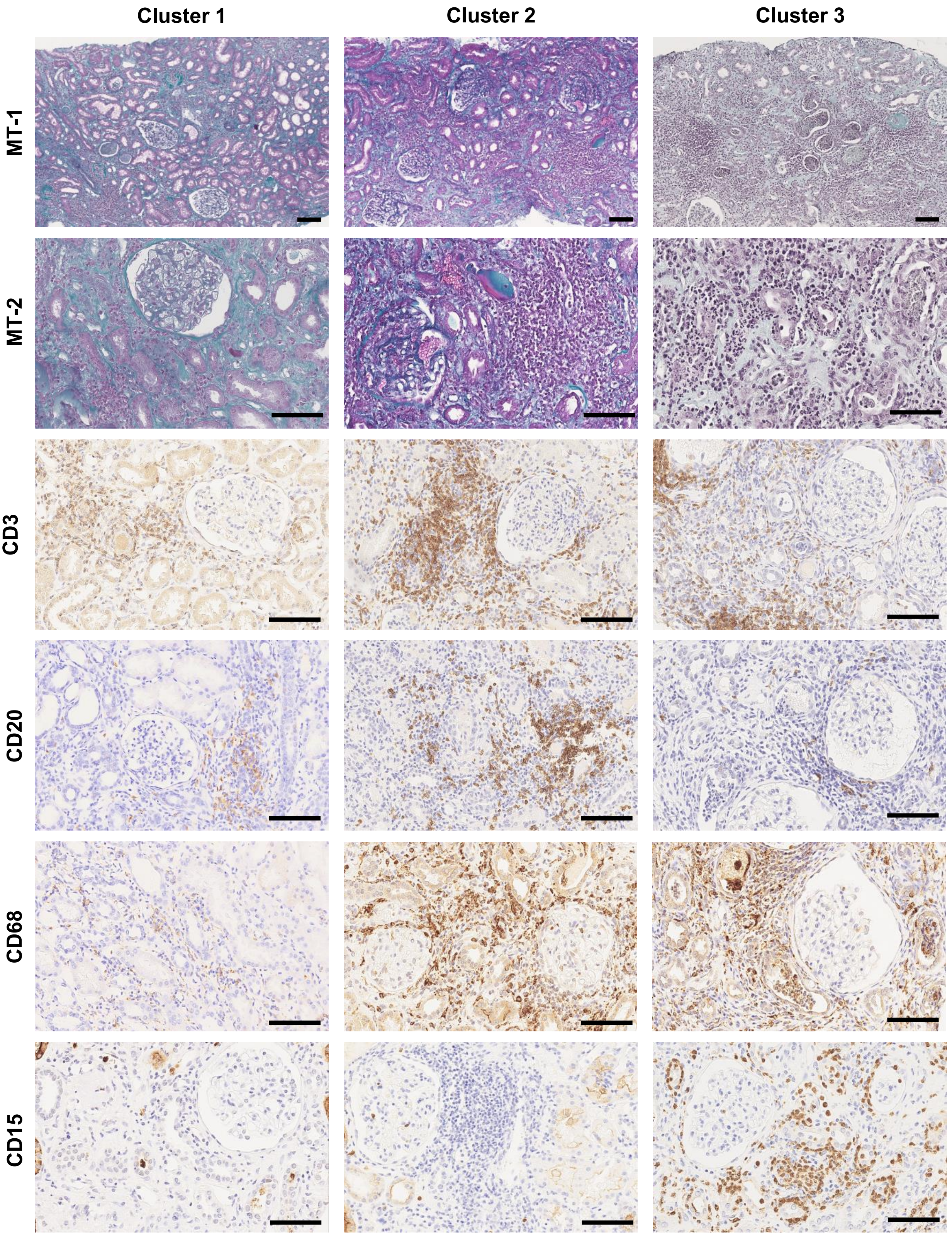

Supplemental Figure 3: Morphological and immunochemistry analysis of ICI-AIN kidney biopsies. MT1 and 2: Representative images of KB using light microscopy with Masson's trichrome stain (MT) from each of the three identified clusters. **CD3, CD20, CD68 and CD15**: Representative images of IHC staining for: CD3, CD20, CD68 and CD15. Scale bars = 100µm

Supplemental Figure 4: Example of AIN recurrence in a patient from cluster 3, occurring six months after immunotherapy discontinuation and corticosteroid cessation.

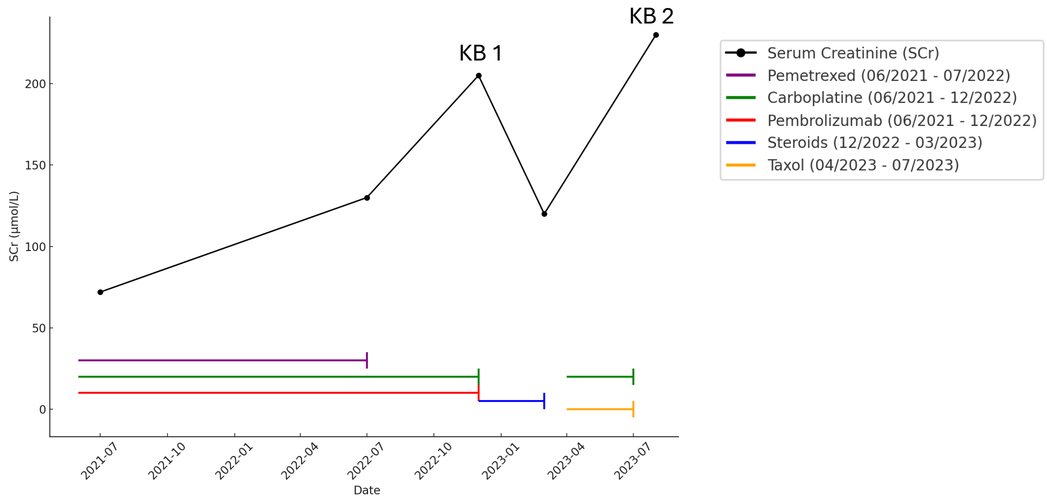

KB 1

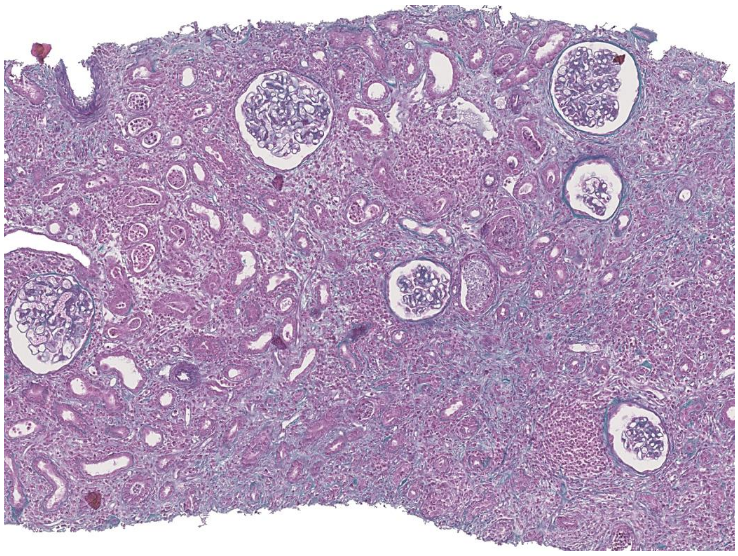

KB 2

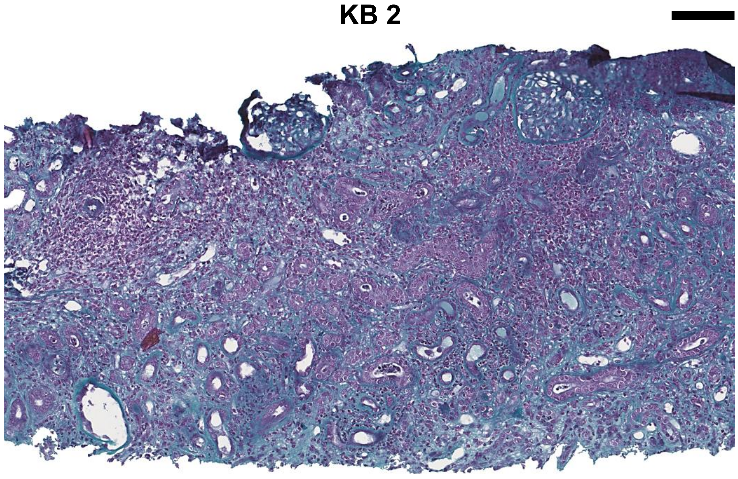

The recurrence exhibited a similar pattern of neutrophil-rich infiltrate, characterized by numerous neutrophils, neutrophilic tubulitis, and granular casts. Scale bar: 100 µm; original magnification: ×20

Supplemental Figure 5. Evolution of the neutrophil-to-lymphocyte ratio (NLR) over time in Cluster 3 according to relapse status.

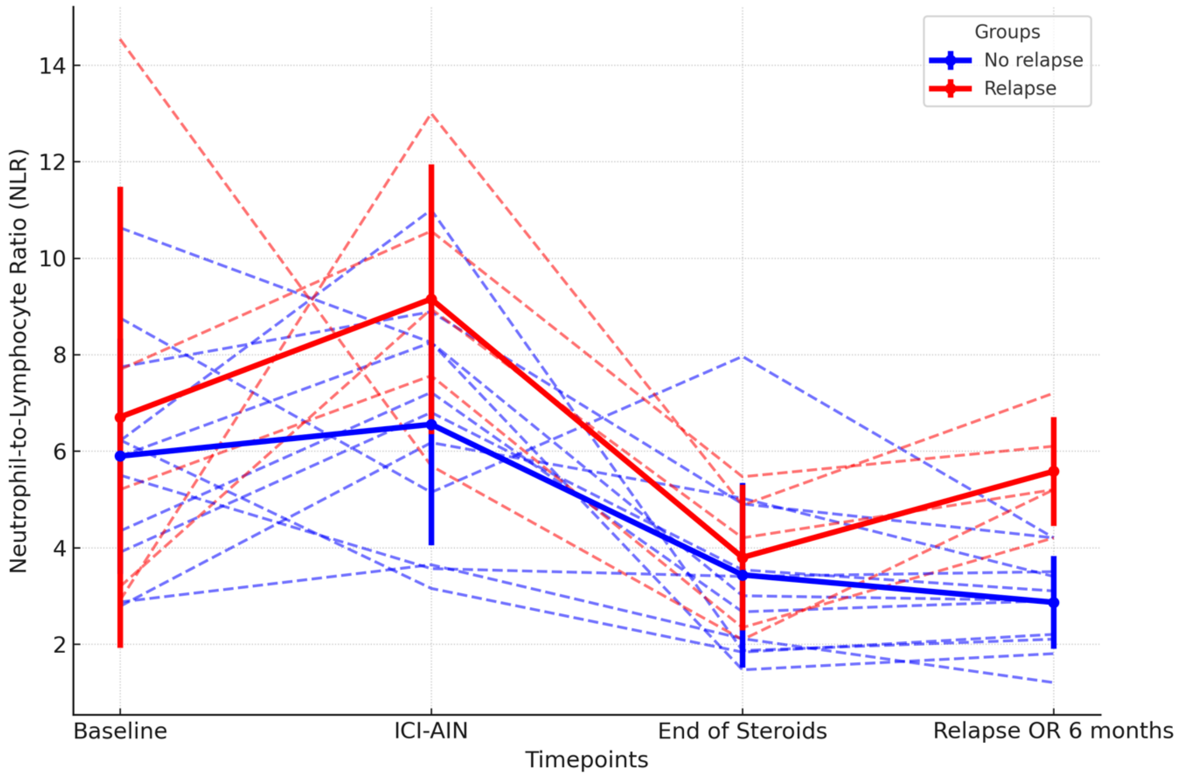

Longitudinal changes in NLR were assessed at baseline (immunotherapy initiation), at the time of ICI-AIN diagnosis, at the end of steroid therapy, and at relapse or 3–6 months after steroid initiation in patients without relapse. Individual trajectories are shown as dashed lines, and group means ± SEM are indicated by solid lines (red for relapse, blue for no relapse). NLR tended to be higher during the ICI-AIN episode in patients who subsequently relapsed and increased again at the time of relapse, compared with patients who did not experience relapse.

STROBE Statement—checklist of items that should be included in reports of observational studies

	Item No	Recommendation	Page/line
**Title and abstract**	1	(*a*) Indicate the study's design with a commonly used term in the title or the abstract	1
		(*b*) Provide in the abstract an informative and balanced summary of what was done and what was found	2
**Introduction**			
Background/rationale	2	Explain the scientific background and rationale for the investigation being reported	5 line 101-110
Objectives	3	State specific objectives, including any prespecified hypotheses	Pahge 5
**Methods**			
Study design	4	Present key elements of study design early in the paper	6
Setting	5	Describe the setting, locations, and relevant dates, including periods of recruitment, exposure, follow-up, and data collection	7
Participants	6	(*a*) *Cohort study*—Give the eligibility criteria, and the sources and methods of selection of participants. Describe methods of follow-up<br>*Case-control study*—Give the eligibility criteria, and the sources and methods of case ascertainment and control selection. Give the rationale for the choice of cases and controls<br>*Cross-sectional study*—Give the eligibility criteria, and the sources and methods of selection of participants	8
		(*b*) *Cohort study*—For matched studies, give matching criteria and number of exposed and unexposed<br>*Case-control study*—For matched studies, give matching criteria and the number of controls per case	
Variables	7	Clearly define all outcomes, exposures, predictors, potential confounders, and effect modifiers. Give diagnostic criteria, if applicable	9
Data sources/ measurement	8*	For each variable of interest, give sources of data and details of methods of assessment (measurement). Describe comparability of assessment methods if there is more than one group	*9*
Bias	9	Describe any efforts to address potential sources of bias	9
Study size	10	Explain how the study size was arrived at	11
Quantitative variables	11	Explain how quantitative variables were handled in the analyses. If applicable, describe which groupings were chosen and why	9
Statistical methods	12	(*a*) Describe all statistical methods, including those used to control for confounding	9
		(*b*) Describe any methods used to examine subgroups and interactions	9
		(*c*) Explain how missing data were addressed	9
		(*d*) *Cohort study*—If applicable, explain how loss to follow-up was addressed<br>*Case-control study*—If applicable, explain how matching of cases and controls was addressed<br>*Cross-sectional study*—If applicable, describe analytical methods taking account of sampling strategy	
		(*e*) Describe any sensitivity analyses	

Continued on next page

**Results**			
Participants	13*	(a) Report numbers of individuals at each stage of study—eg numbers potentially eligible, examined for eligibility, confirmed eligible, included in the study, completing follow-up, and analysed	11
		(b) Give reasons for non-participation at each stage	11
		(c) Consider use of a flow diagram	11
Descriptive data	14*	(a) Give characteristics of study participants (eg demographic, clinical, social) and information on exposures and potential confounders	11
		(b) Indicate number of participants with missing data for each variable of interest	11
		(c) *Cohort study*—Summarise follow-up time (eg, average and total amount)	11
Outcome data	15*	*Cohort study*—Report numbers of outcome events or summary measures over time	*15*
		Case-control study—Report numbers in each exposure category, or summary measures of exposure	*15*
		Cross-sectional study—Report numbers of outcome events or summary measures	
Main results	16	(*a*) Give unadjusted estimates and, if applicable, confounder-adjusted estimates and their precision (eg, 95% confidence interval). Make clear which confounders were adjusted for and why they were included	11-17
		(*b*) Report category boundaries when continuous variables were categorized	11-17
		(*c*) If relevant, consider translating estimates of relative risk into absolute risk for a meaningful time period	
Other analyses	17	Report other analyses done—eg analyses of subgroups and interactions, and sensitivity analyses	11-17
**Discussion**			
Key results	18	Summarise key results with reference to study objectives	18
Limitations	19	Discuss limitations of the study, taking into account sources of potential bias or imprecision. Discuss both direction and magnitude of any potential bias	20
Interpretation	20	Give a cautious overall interpretation of results considering objectives, limitations, multiplicity of analyses, results from similar studies, and other relevant evidence	18
Generalisability	21	Discuss the generalisability (external validity) of the study results	20
**Other information**			
Funding	22	Give the source of funding and the role of the funders for the present study and, if applicable, for the original study on which the present article is based	21

*Give information separately for cases and controls in case-control studies and, if applicable, for exposed and unexposed groups in cohort and cross-sectional studies.

Note: An Explanation and Elaboration article discusses each checklist item and gives methodological background and published examples of transparent reporting. The STROBE checklist is best used in conjunction with this article (freely available on the Web sites of PLoS Medicine at http://www.plosmedicine.org/, Annals of Internal Medicine at http://www.annals.org/, and Epidemiology at http://www.epidem.com/). Information on the STROBE Initiative is available at www.strobe-statement.org.
